# simpleISM—A straight forward guide to upgrade from confocal to ISM

**DOI:** 10.1371/journal.pone.0279378

**Published:** 2022-12-27

**Authors:** Monalisa Goswami, René Lachmann, Robert Kretschmer, Rainer Heintzmann

**Affiliations:** 1 Leibniz Institute of Photonic Technology, Jena, Germany; 2 Institute of Physical Chemistry and Abbe Center of Photonics, Friedrich-Schiller-University, Jena, Germany; Goa University, India, INDIA

## Abstract

Resolution in a confocal laser scanning microscopes (CLSM) can be improved if the pinhole is closed. But closing the pinhole will deteriorate the signal to noise ratio (SNR). A simple technique to improve the SNR while keeping the resolution same by upgrading the system to an image scanning microscope. In this paper, we explain in detail, based on an Olympus Fluoview 300 system, how a scanning microscope can be upgraded into an image scanning microscope (ISM) using a simple camera-based detector and an Arduino Due providing a galvo driving and camera synchronization signals. We could confirm a resolution improvement as well as superconcentration and made the interesting observation of a reduced influence of laser fluctuations.

## Introduction

Confocal microscopes are popular microscopes among biologists mainly because of their exceptional sectioning capability. By placing a field stop (pinhole) in front of the detector, most of the out-of-focus light from the thick samples, which contributes to the image blur, gets rejected. At typically used pinhole sizes, the images have good contrast but the resolution remains essentially unaltered compared to a widefield microscope. To achieve a significant improvement in resolution, the pinhole must be closed down to an extent (<0.3 Airy Unit (AU)) [1] that most of the light gets discarded. As a result, although the resolution improves by a factor of $\sqrt{2}$ (assuming a Gaussian point spread function) compared to a widefield image, the signal to noise ratio reduces drastically.

In 1982, Sheppard [2] and colleagues discovered that the resolution of a confocal microscope improves if the detector pinhole is shifted (or offset) over the first dark ring in the Airy disk at the detector plane. This is the principle behind image scanning microscopy (ISM) [3, 4]. In ISM, the confocal microscope is modified such that the pinhole with a bucket detector (e.g. a PMT) is replaced by an array detector (like a CCD camera or a SPAD array or a PMT array) and the pinhole is removed to detect all available fluorescence. The fluorescence collected from each scan position is recorded as a series of frames (in case of a camera) and then reconstructed into one super-resolution image using pixel-reassignment. The result is an improvement in resolution equivalent to a closed pinhole confocal configuration (<0.3 AU) but without discarding photons and hence exhibit higher signal to noise ratio.

**Fig 1:**
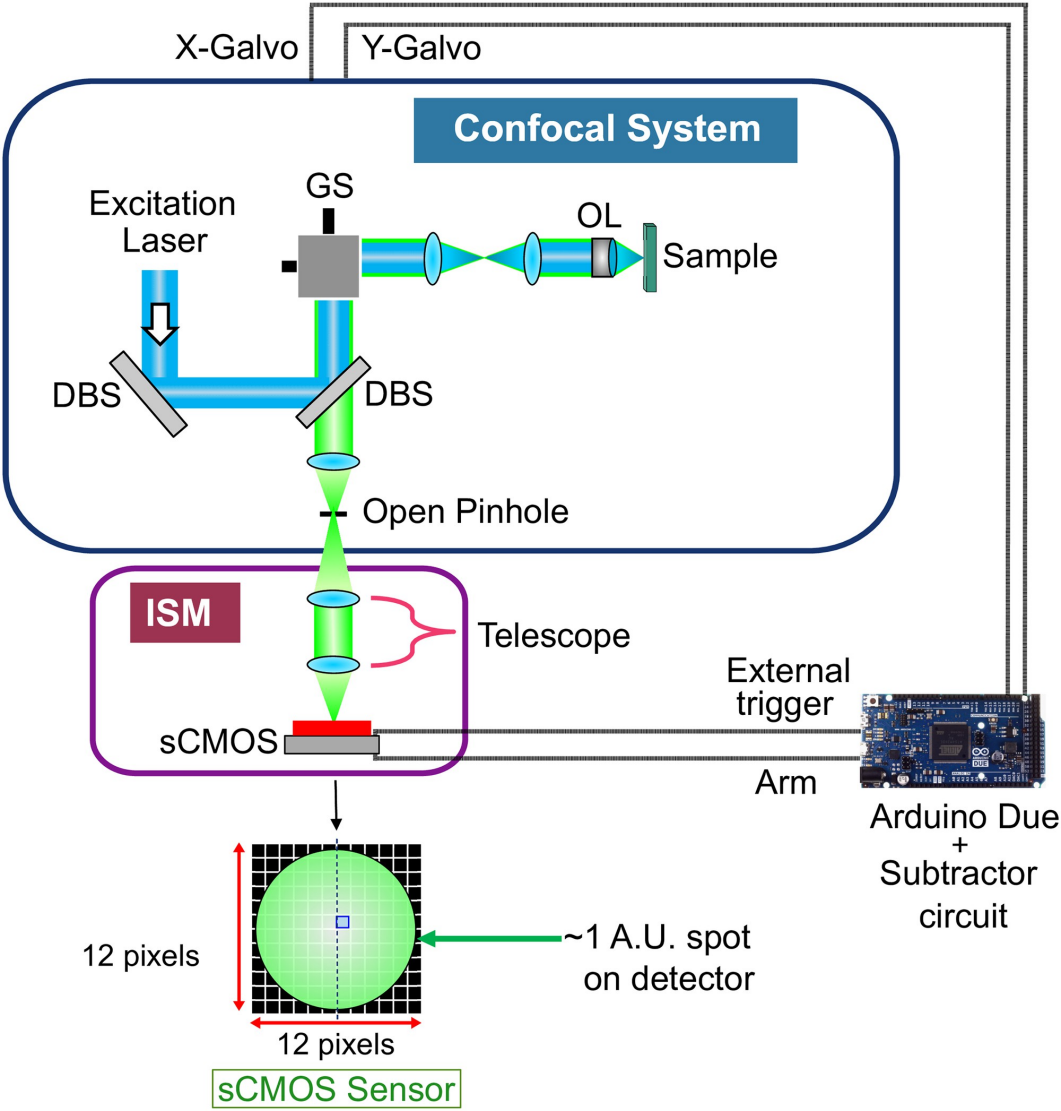
Image scanning microscope (ISM) setup. The excitation laser beam is reflected from the dichromatic beam splitters (DBS) and the galvanometric scanner (GS) and is focussed onto the sample by the objective lens (OL). The reflected fluorescence is collected by the same OL, is descanned and reflected towards the detector via the pinhole. ‘Open pinhole’ is the position of the pinhole where the pinhole diameter was maximal to collect all available fluorescence. Here, the bucket detector (PMT) of a conventional confocal microscope is replaced by an array detector (Andor Neo sCMOS camera) and the telescope is used in the detection arm to image the pinhole plane onto the camera. The magnification of the telescope is chosen such that the 12 × 12 pixels sub-array on the camera will correspond to approximately 1 AU. An Arduino Due with a subtractor circuit is used to provide the synchronization between the camera and the galvanometric mirrors.

While a very good guide to constructing a spinning-disc-based ISM version [5] has been published, we here describe an easy method to transform an Olympus Fluoview-300 IX71 confocal laser scanning microscope (CLSM) into a high resolution image scanning microscope. Fig 1 shows the experimental setup.

The bucket detector (PMT) of a conventional confocal microscope is replaced by an array detector (Andor Neo sCMOS camera) and the telescope is used in the detection arm to image the pinhole plane onto the camera. The magnification of the telescope is such that 12 × 12 pixels on the camera correspond to approximately 1.0 AU (diameter of an Airy disk) spot size resulting in the pixel size of 0.08 AU. Hence, each pixel will behave as a detector with almost closed pinhole (pinhole with smallest diameter). The pinhole, albeit still be present in the microscope, was kept open (to the maximum pinhole diameter position) to detect all available fluorescence.

Although deceptively simple, there is one major challenge to this upgrade: to match the high scan speed of the galvanometric mirrors (lines per second) to the frame rates of the camera. In the firmware of this system, there is a lower limit to the scan speed (256 lines per second). This means that to achieve Nyquist sampling, more than 131,000 camera frames per second would need to be acquired. An additional challenge is a proper synchronization of the frame acquisition to the pixel clock of the scan. Choosing a smaller region-of-interest (ROI) can increase the frame rates in the camera. For this reason, the smallest possible ROI of 12 × 12 pixels was chosen on the Andor Neo sCMOS camera which yielded a maximum frame rate of only ≈1900 frames per second. This limitation in the frame rate comes from the camera link connector which slows down the interfacing between the camera and the computer in external-trigger mode. Therefore, the only way to synchronize the fast moving galvanometric mirrors with the camera is to slow down the scan speed, for the pixel clock to match the available acquisition speed of the camera. This synchronization was achieved by driving the system in a slow scanmode mode via an Arduino Due.

The reason for choosing an Arduino Due is it has two DAC (Digital to Analog converter) pins that can provide the necessary scan voltages required by the galvanometric mirrors. Pin 51 of the Arduino Due is used to provide a 5V TTL (transistor-transistor logic) trigger signal to the “external trigger” input of the camera. The TTL timing output from the “ARM” of the camera is provided as an input to the pin 53 of the Arduino Due. During acquisition, the ARM will be LOW indicating that no trigger event will be accepted during this period. The Arduino waits until the ARM is HIGH again to provide the camera with the next trigger signal. After each acquisition, a scan voltage signal is sent to the galvanometric mirrors in a ramp-like fashion. The range of the linear ramp voltage decides the field-of-view (FOV) in the sample plane.

**Fig 2:**
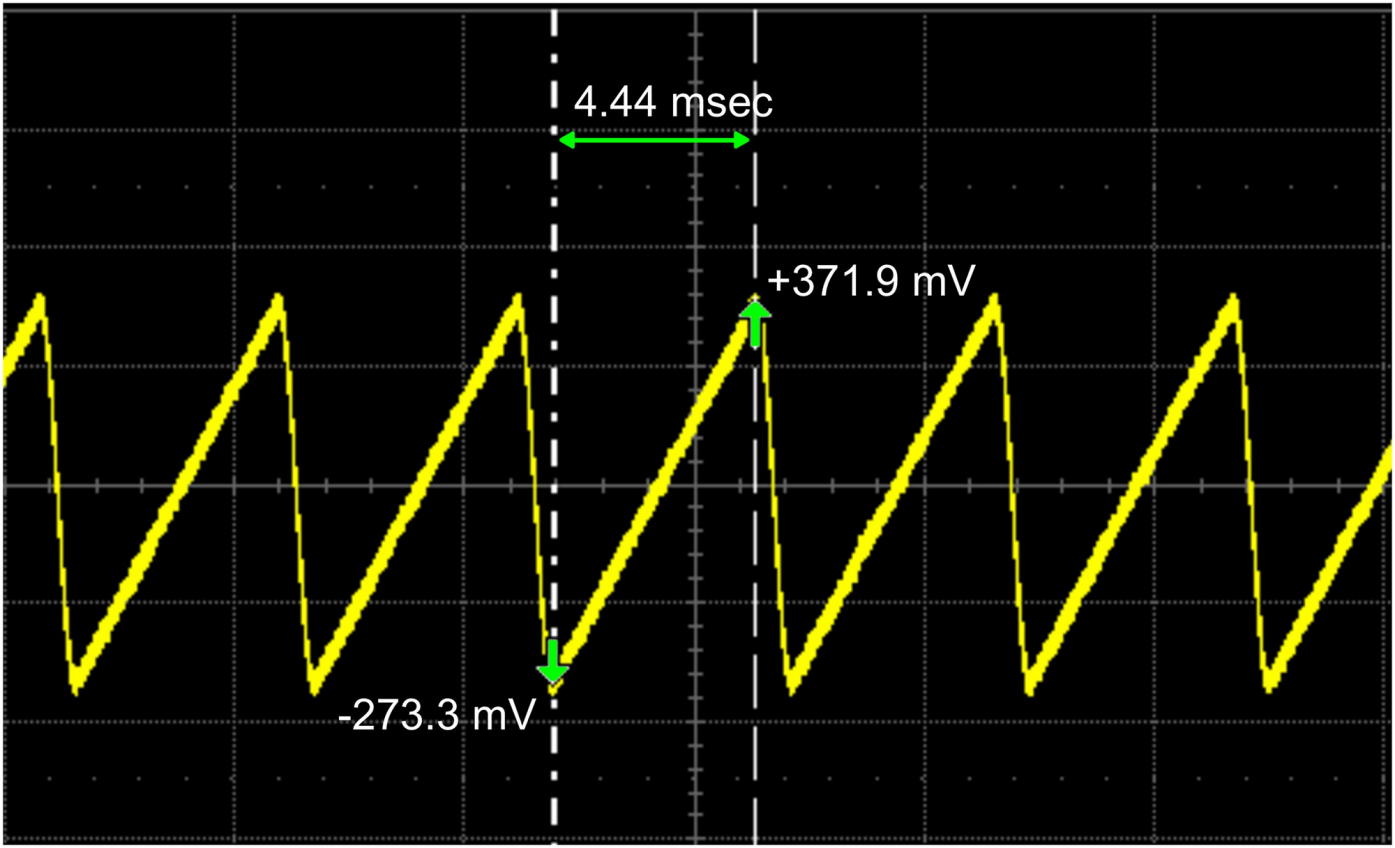
Line scan voltages from the Fluoview software as observed on the oscilloscope. Pin 11 (for x-galvanometric mirror) of the DB-15HD connector at the back of the Fluoview computer was connected to the oscilloscope. The peak to peak voltage (-273.3 mV to +371.9 mV) determines the galvanometric mirror movement and hence the scan area in the sample plane. The period of the ramp signal (4.44 msec) is the time required to scan each line. A similar ramp signal (not shown here) was observed from pin 15 (constituting y-galvanometric mirror) of the connector but with a significantly longer pulse duration.

To determine the ratio between the scanned sample distance (field of view) and the scanned amplitude voltage (Vpp) of the ramp signal, we intercepted two pins (pin 11 and 15) of the DB-15HD connector situated between the board (Fluoview computer) and the power supply unit (PSU) of the confocal microscope, and observed it on the oscilloscope. Pin 11 generates the voltage signal for pixel scan (x-galvanometric mirror) and pin 15 generates the voltage signal for line scan (y-galvanometric mirror). Fig 2 shows the signal coming from pin 11 on an oscilloscope.

The protocol described in this peer-reviewed article is published on protocols.io, https://dx.doi.org/10.17504/protocols.io.kxygx9mz4g8j/v1 and is included for printing as S1 File with this article.

The measurements were performed in “*Continuous-scan*” mode on the Fluoview software. A scan size of 512 × 512 pixels was selected at zoom 10 to satisfy the Nyquist sampling condition of λ_*excitationl*_/8NA for CLSM [6]. For an excitation wavelength of 488 nm and a 1.4NA oil immersion objective, the correct sampling (or pixel pitch) is ≈ 43 nm which is close to the sampling (46 nm) we chose for the above mentioned scan size and zoom. In Fig 2, the Vpp from Fluoview software varies from -273.3 mV to +371.9 mV which represents the voltage range for one line scan in the sample plane. Each line consists of 512 pixels giving the pixel shift voltage (or the step size of the ramp signal) of

$$\begin{array}{c} P_{shift}=\left(\left(371.9\text{mV}-\left(-273.3\text{mV}\right)\right)/512\text{pixels}\right)\approx 1.26\text{mV}/\text{pixel} \end{array}$$ (1)

**Fig 3:**
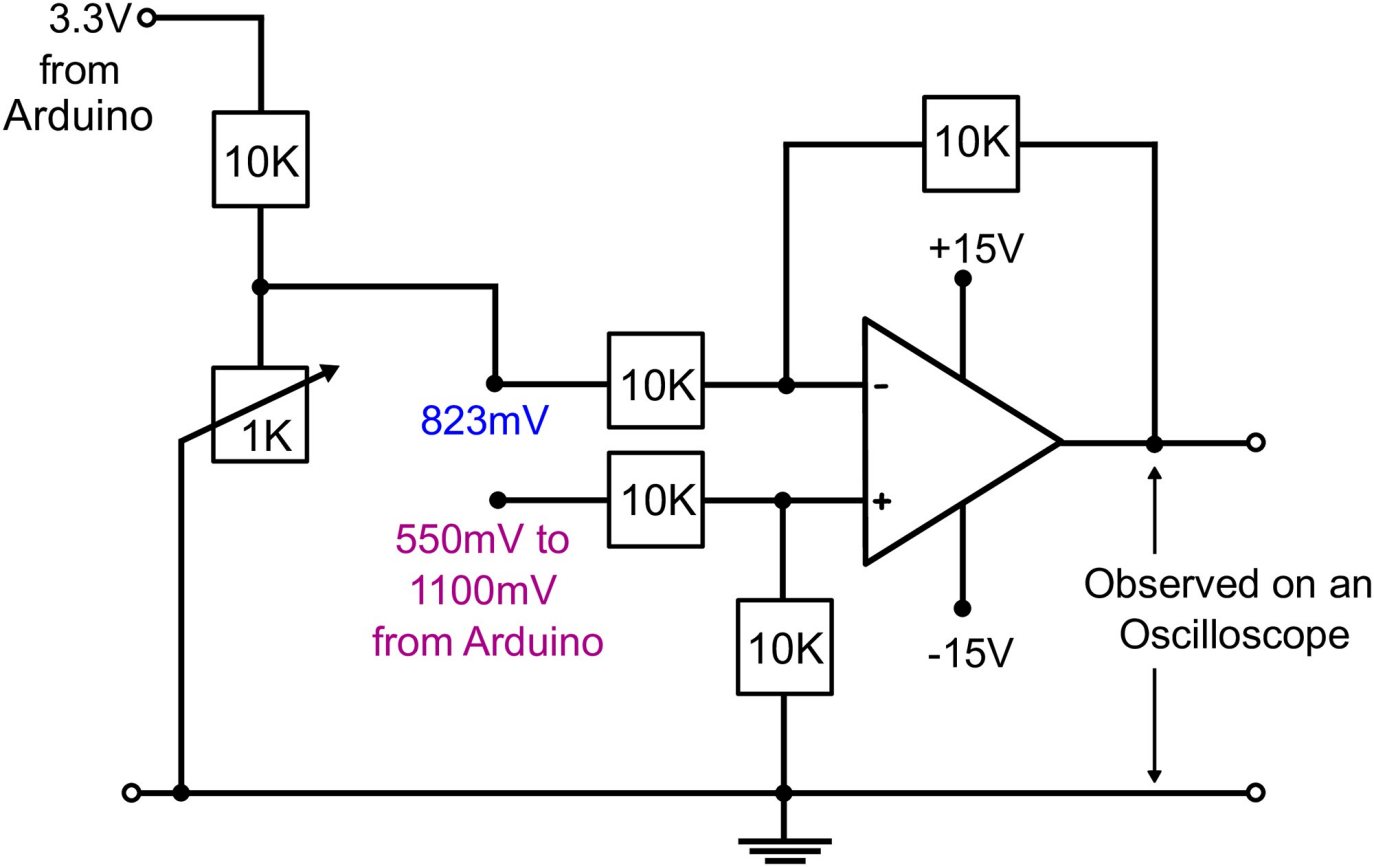
Subtractor circuit. It is essentially a differential amplifier (op-amp) with a ramp voltage input (550 mV to 1100 mV) from the DAC pin of Arduino provided at the one input pin of the op-amp and the shift voltage signal (823 mV)) to the other input pin. To fine tune the shift voltage, a variable resistor is used.

The output voltage from the two DAC pins of Arduino Due ranges from +550 mV to +2.75 V. To match the FOV for the given *P*_*shift*_ voltage, the Arduino Due was operating at 11-bit DAC resolution and the output voltage is sent to a subtractor circuit (Fig 3) to shift the voltage range by approximately 823 mV.

**Fig 4:**
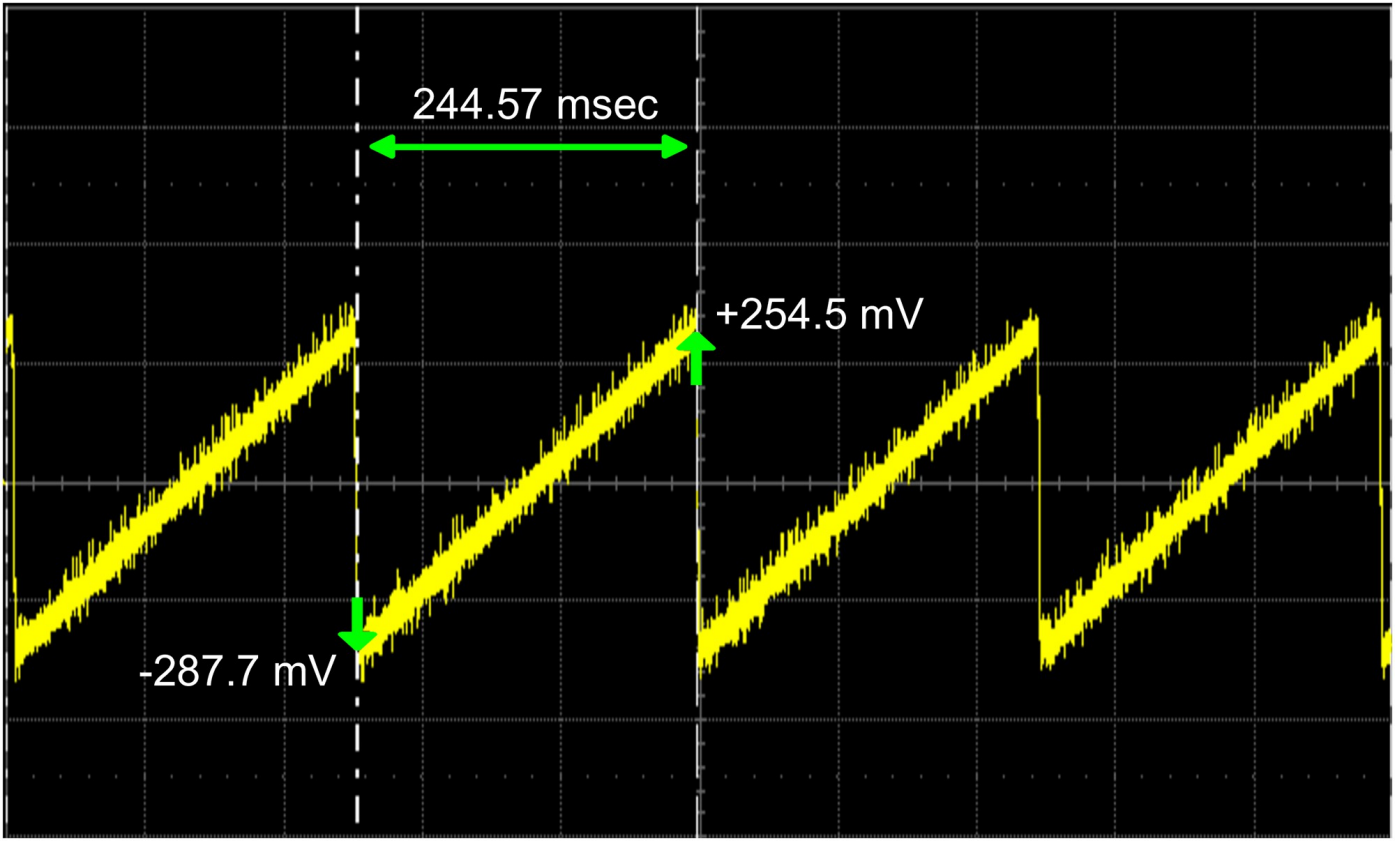
The output voltage signal from the Arduino Due via subtractor circuit observed on an oscilloscope. When compared to Fig 2, it is clear that the Vpp (-287.7mV to +254.5 mV) approximately matches but the pulses now has a longer duration (244.57 msec). This is because the galvanometric mirrors now scans at the same (slow) speed as that of the frame rate of camera.

Fig 4 shows the resultant voltage range as observed on the oscilloscope. Our obtained range of scan voltages (-287.7 mV to +254.5 mV) was slightly smaller than the one obtained from the Fluoview software, but this small mismatch has no negative influence on the performance.

**Fig 5:**
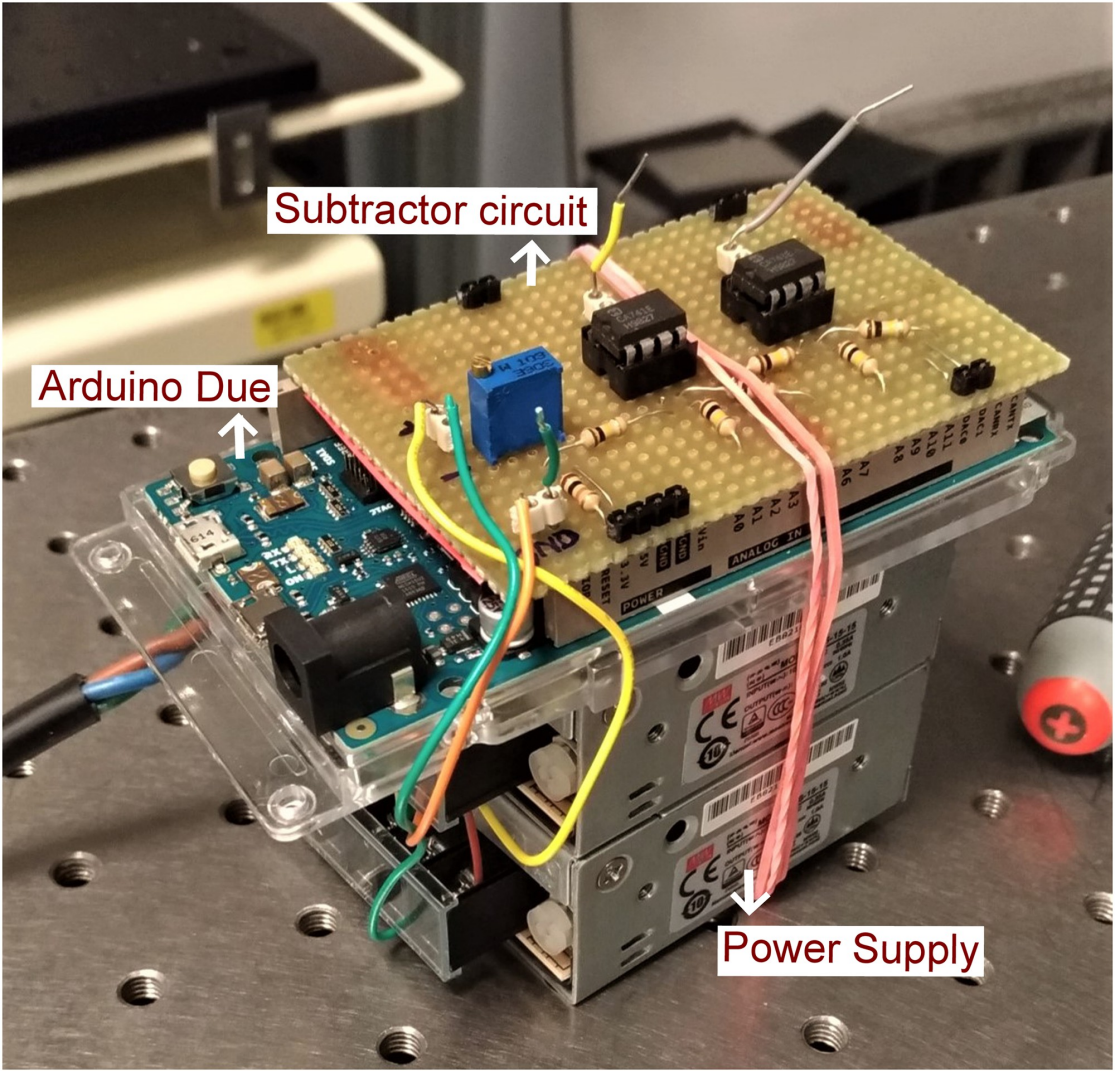
Subtractor circuit with Arduino Due.

Fig 5 shows the subtractor circuit with the Arduino Due.

To acquire ISM data, a series of images were captured on the camera using the following settings (Table 1) in the software (Andor Solis) of the camera.

**Table 1:** Software settings on Andor Neo sCMOS camera.

Acquisition mode	Kinetic series
Electronic shuttering mode	Rolling
Kinetic series length	262,144
Exposure time	0.00040 seconds

It is important to note here that only the scan voltages to the galvanometric mirrors are changed and are provided by the Arduino Due, the rest of the microscope, including the power supply to the galvanometric mirrors, is still controlled by the Fluoview software. Hence, during the ISM measurement, the Fluoview software should be running in “continuous-scan” mode.

**Fig 6:**
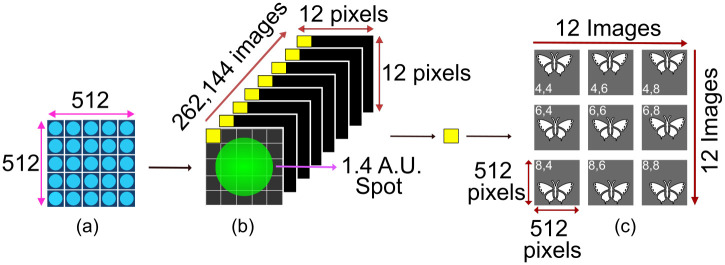
Basic image reconstruction from the acquired camera frames. (a) The laser (blue dots) scans 512 × 512 positions at the sample plane. (b) For each scan position, the camera captures a corresponding frame of 12 × 12 pixels. The yellow boxes represents pixels. (c) One such pixel from each frame is taken and arranged in a 512 × 512 fashion to form an image. Depending upon the pixel position in the frames, the image will be slightly shifted with respect to the image reconstructed from central pixel. The numbers in the image represents the pixel number. Summing all the images will results in an open pinhole confocal image.

The data acquisition and the basic image reconstruction scheme is explained in Fig 6. The excitation laser scans the sample and the camera acquires a corresponding 4D-image-stack consists of 12 × 12 pixels for each of the 512 × 512 scan positions and hence, in total 12 × 12 × 512 × 512 pixels. These pixels were arranged in a 512 × 512 fashion to form an image. The data on the camera is saved in the “.fits” format to reduce the file size and later, to speed up the reconstruction process (in MATLAB).

Summing all the images in software (MATLAB) results in a confocal image with an open pinhole (termed as Open Confocal in this paper). The image obtained by choosing only the central acquisition pixel represents an approximation to the ideal closed confocal image (Closed Confocal). To achieve an ISM image, following reconstruction scheme is used.

## Basic ISM reconstruction schemes

A simple forward model to describe the measured 4D data set can be described via the imaging equation

$$\begin{array}{c} M_{m,n}(\vec{x})=[h_{m,n}⊗S](\vec{x}) \end{array}$$ (2)

where *M* is the measured 3D image stack, *h* is the 3D intensity point spread function (PSF) of the microscope, *m*, *n* the detector/pixel indices, *S* is the 3D sample and $\vec{x}=\left(x,y,z\right)$ the 3D coordinate vector in the image space. Note that $\vec{x}$ and image *M* only have discrete values while the convolution ⊗ (in the sample plane), the PSF and the sample are continuous. Each pinhole has a slightly different viewing angle of the sample and thus measures a slightly different 3D volume in the sample, allowing more information about the sample to be obtained. Thus, classical methods, such as the open confocal (CFo) (Eq 3) or the closed-pinhole confocal (CFc) (Eq 4)

$$M^{\text{(CFo)}}(\vec{x})=\sum_{m,n}M_{m,n}(\vec{x})$$ (3)

$$M^{\text{(CFc)}}(\vec{x})=M_{m_{c},n_{c}}(\vec{x})$$ (4)

with *m* = *m*_*c*_, *n* = *n*_*c*_ the indices of the central pinhole, can be directly regained from the measurement. The “Sheppard sum” [7] models the effective illumination and detection PSF as a Gaussian function, which allows to analytically calculate the most likely emission position of the emitter located in the sample based on the scanning geometry and wavelengths used. It is found that the assigned detector position is further displaced from the excitation position than the most likely emission position, and thus a shift factor can be calculated to correct for this error. This yields the reassigned image $M^{\text{(Reass)}}(\vec{x})$:

$$\begin{array}{c} M^{\text{(Reass)}}(\vec{x})=\sum_{m,n}M_{m,n}\left(x-\Delta x_{m,n},y-\Delta y_{m,n},z\right) \end{array}$$ (5)

where Δ*x*_*m*,*n*_, Δ*y*_*m*,*n*_ are the lateral X- and Y-displacements depending on the detector pixel. In our method we avoid assumptions about the PSF profile of the optical system and the choice of the individually determined shift-vectors per detector pixel automatically accounts for changes in wavelength and non-gaussian shapes. The shifts are determined by means of correlation (⋆):

$$\begin{array}{c} \left(\Delta x_{m,n},\Delta y_{m,n})=\underset{\vec{x}}{\text{argmax}}(M_{m_{c},n_{c}}⋆M_{m,n}\right) \end{array}$$ (6)

where argmax finds for the position $\vec{x}$ where the correlation takes a (global) maximum. To this aim we use DIPimage [8] routine ‘findshift’ in MATLAB toolbox that iteratively maximizes Eq 6 by applying sub-pixel shifts. The correlation (Eq 6) and convolution (Eq 5) are realized in Fourier space and are implemented using Fast Fourier Transformation (FFT). Sub-pixel shifts in the case of Eq 5 can thus be realized without rescaling.

## Calibration of ISM images

**Fig 7:**
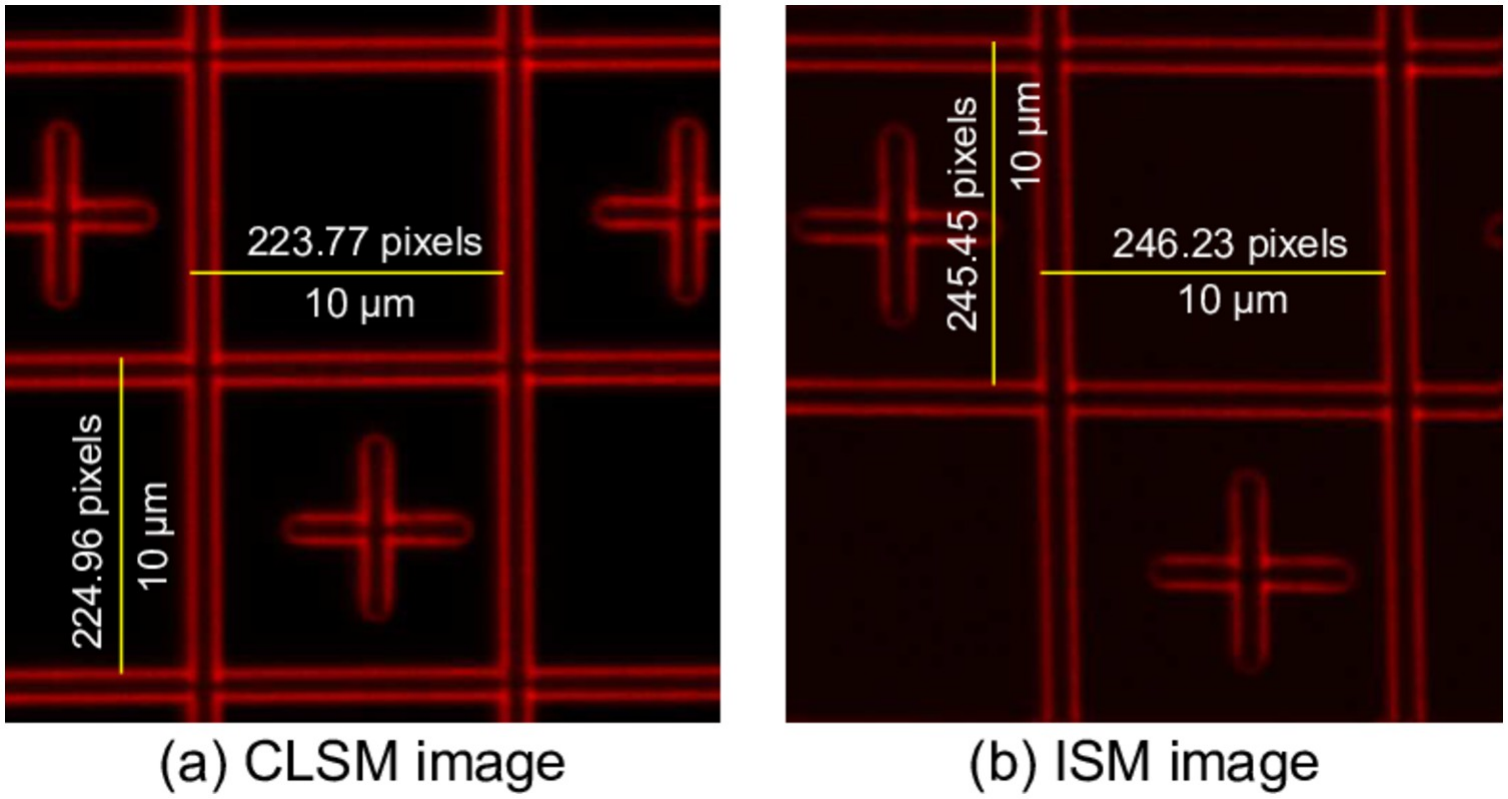
Image of the grid pattern on the Argolight calibration slide. The pixel pitch of the CLSM and ISM images were calculated in both x and y directions using the known pitch of 10 *μm* of each square box. The resultant pixel pitch in case of CLSM is [0.0446*μm*, 0.0444*μm*] and in case of ISM [0.0406*μm*, 0.0407*μm*].

To calculate the pixel pitch, ISM images were investigated using an Argolight calibration slide [9]. The slide contains several fluorescent patterns for a wide range of excitation wavelengths. Fig 7 shows the fluorescence image of one such pattern excited with a 488 nm laser. The pitch of each square in the grid pattern is 10 *μm*.

For the CLSM images, the pixel pitch can be known from the “experimental properties” of the Fluoview confocal firmware. For a scan size of 512 × 512 at the zoom setting 10, the pixel pitch shown by the firmware is 0.0460 *μm*. To cross check this value, a calibration was performed using the Argolight calibration slide (Fig 7a) which resulted in a pixel pitch value of [0.0446*μm*, 0.0444*μm*] which is in reasonable agreement with the one reported by the Fluoview firmware. We therefore used the Argolight calibration slide as our reference and obtained the pixel pitch value of [0.0406*μm*, 0.0407*μm*] (Fig 7b) when the scan was driven by the Arduino.

The difference in the pixel pitch originates from the fact that the *P*_*shift*_ provided by the Arduino Due (1.07 mV) was slightly different from the one provided by the Fluoview software (1.26 mV). The pixel pitch of ISM will be used to calculate the improvement in resolution in the upcoming section.

## Results

To observe an improvement in resolution and SNR, the point spread function (PSF) of the system was measured. Sub-nanometer sized fluorescent beads (Gattabeads: OG-488, 23 nm diameter) were illuminated with a 488 nm laser (Omicron LuxX.HSA 488–150).

For a fair comparison, we compared the reconstructed open confocal image with the ISM image instead of using the CLSM image. The reason is that the CLSM requires approximately 2 seconds to scan a sample or to take an image whereas ISM takes roughly 2 minutes to scan a complete sample. Since the pixel dwell time in case of ISM was more, to avoid bleaching, the excitation laser power that was used on the sample was much less as compared to the CLSM. And because of the different laser power used, we will instead compare the reconstructed open confocal images with the ISM images. The closed confocal image is obtained by only choosing the central acquisition pixel and arranging it in a 512 × 512 fashion.

To calculate the PSF, 66 beads at sufficient distance from their neighbours were selected and a ROI of 12 × 12 pixels was extracted around each bead. The maximum position of each bead is determined using ‘findshift’ (DIPimage [8]) and the individual PSFs are shift corrected and summed. A Gaussian fit was used to determine the FWHM (Full Width at Half Maximum) of the PSF.

**Fig 8:**
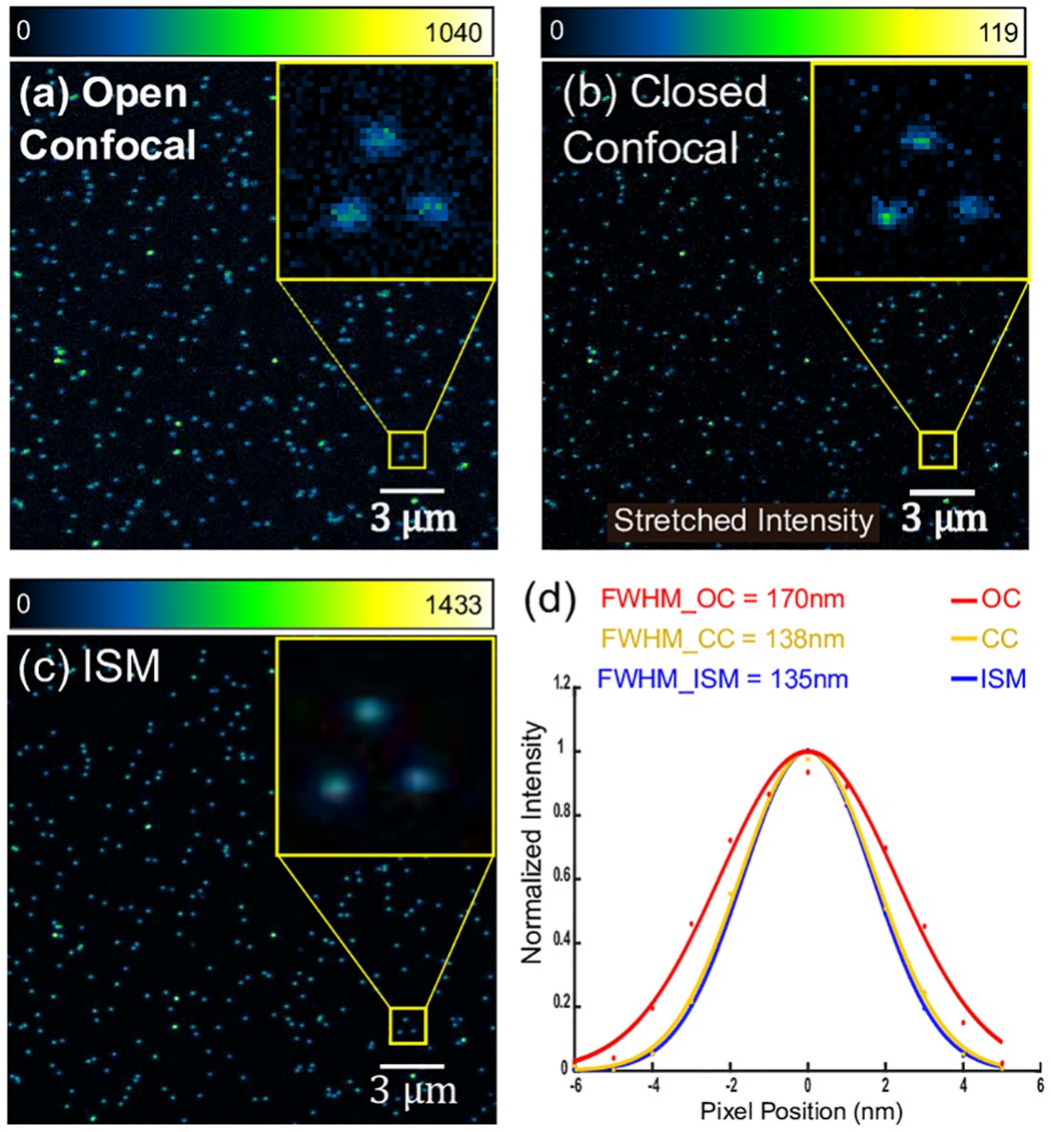
PSF measurement using sub-diffraction size beads. (a) Open confocal image is obtained by summing the raw images from all the pixels. (b) Closed confocal image is obtained by choosing the central acquisition pixel. The intensities are stretched for better visualization. (c) ISM image is obtained by using “Sheppard sum”. All panels were zero-clipped after background subtraction for better visualization. (d) The plot shows PSF obtained by taking a Gaussian fit. The reduced width of ISM is apparant when compared to open confocal. There is also a slight improvement in PSF of ISM when compared to closed confocal.

As seen from Fig 8, the FWHM in case ISM is 135 nm which is 1.25× better than the FWHM of 170 nm observed in case of open confocal and roughly the same as the FWHM of 138 nm observed in case of closed pinhole configuration. The high NA simulations yielded a PSF of 168 nm for open confocal which is in close agreement to the experimentally observed value. It is important to note here that the open pinhole confocal is still a confocal image and not a widefield image because the pinhole is not completely removed, plus the excitation is focussed/structured illumination and not widefiled illumination. Hence, an improvement of 1.35 with respect to open confocal is comparable to 1.41 ($\sqrt{2}$) with respect to widefield.

ISM image not only shows a significant improvement in signal (about 1433 ADUs) when compared to closed confocal image (119 ADUs) but also the maximal signal is higher when compared to open confocal image (1040 ADUs). This phenomenon is called “superconcentration” of light [10].

**Fig 9:**
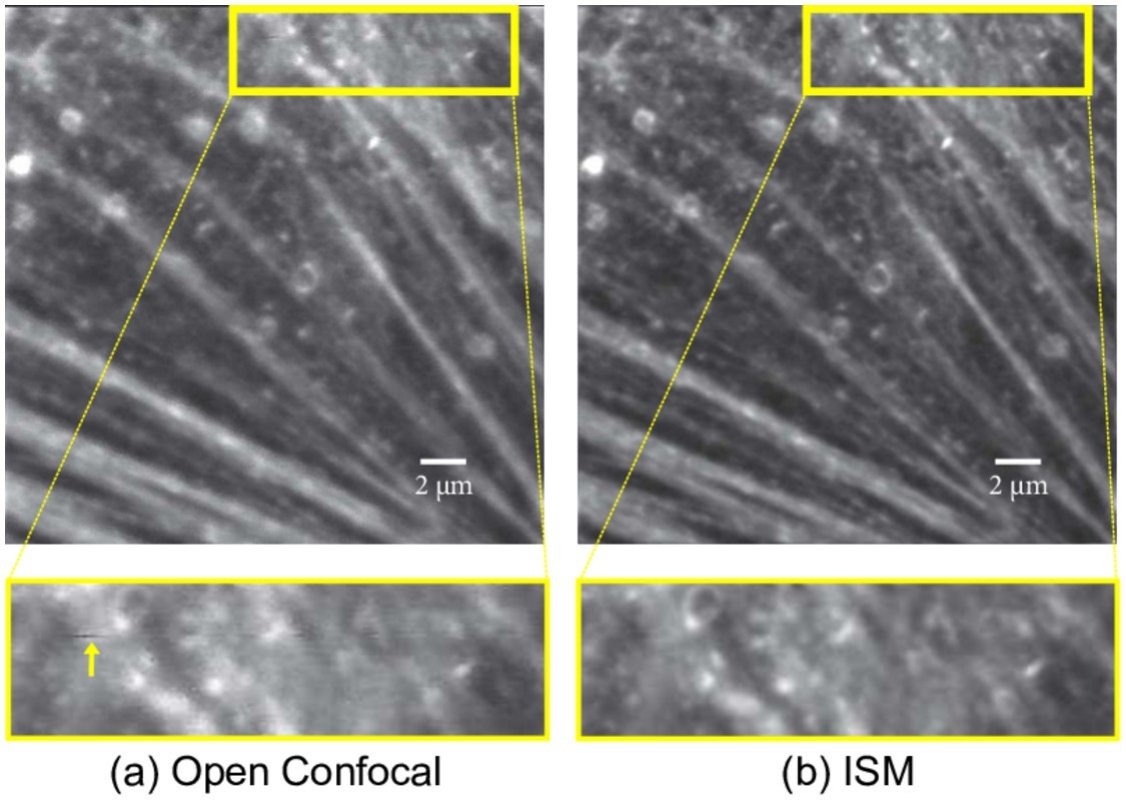
F-actin imaged in BPAE cells. (a) Top figure is an open confocal image and buttom figure is the inset showing laser fluctuation and lower resolution. (b) reconstructed ISM image. The laser fluctuations are inherently mitigated in ISM.

We also imaged some biological samples. Fig 9 shows BPAE (Bovine Pulmonale arterielle Endothelzellen) cells excited with 488 nm laser and 1.4NA oil immersion objective to image F-actin. The area inside the yellow box highlights the laser intensity fluctuation in open confocal image. Pixel reassignment inherently provides time averaging which helps mitigating the undesirable fluctuations in the laser [11]. Even though various applications may require individual choices of objectives, the principle of image scanning microscopy, as demonstrated here, is always applicable. Similar relative improvements in image resolution can be expected as long as both, the excitation and the emission point spread functions, are of similar quality. This means that for a fluorescent dye with a very large Stokes shift (e.g. Chlorophyll IIA being excited at 420nm and detected at 690nm) only a minor relative resolution improvement, compared to open pinhole imaging, of about

$$\begin{array}{c} \frac{1}{420nm\sqrt{\frac{1}{{420nm}^{2}}+\frac{1}{{620nm}^{2}}}}≅0.85 \end{array}$$ (7)

can be expected. Similarly, only a minor resolution improvement with respect to the widefield emission PSF is to be expected, if the illumination beam in the confocal microscope significantly underfills the objective pupil, leading to a low-quality excitation PSF. To avoid bad performance, confocal systems are typically designed to overfill most objective pupils with the collimated illuminating laser beam. Yet, even in case of underfilling the objective pupil by the laser, a PSF of the ISM system would still be significantly better than confocal imaging in such a system with an open pinhole. Overall the ISM is thus expected to perform well for most practical applications.

## Conclusion

In this article, we presented a straight forward way to convert an off the shelf confocal microscope (Olympus Fluoview 300) into an ISM microscope. The main difficulty was the synchronization and interfacing between the system control software and galvanometric scanning and camera acquisition. Our solution was based on intercepting and replacing the galvanometric drive signal and synchronizing it with the camera acquisition with the help of an Arduino Due board. Our implementation using a sCMOS camera allowed only one image (262,144 pixels) per two minutes which is not well suited for live-cell imaging of fast cellular events. We expect that this can be improved by using faster detectors such fiber coupled PMT arrays or using multi-spot scanners. One can also use the cameras from other manufacturers provided they are externally triggerable, and can send back the frame synchronization signal after the frame acquisition is done. In general, a high frame rate camera is preferred. To use the microscopes from other manufacturers, one has to find out the details of the trigger signals and wiring diagrams which may vary from manufacturer to manufacturer. Recently, a hands on guide of converting a spinning disk confocal microscope into ISM is nicely demonstrated achieving impressive scan rate [5]. Although multi-spot scanners are much better suited to achieve high rates, they are expected to have a compromised signal to noise ratio for thick specimen when imaging non-sparsely labelled fluorescence due to pinhole cross-talk. While our system maybe slow, it should still be possible to image rather thick samples due to its single point scanning mechanism. We experimentally confirmed the resolution improvement and superconcentration in beads test samples as well as on biological samples.

## Supporting information

S1 File Uploaded in the system. (PDF)
