# Supplementary material for: simpleISM—A straight forward guide to upgrade from confocal to ISM
Source: PLoS One. 2022 Dec 27;17(12):e0279378. doi: 10.1371/journal.pone.0279378 (PMC9794089; doi:10.1371/journal.pone.0279378)
Supplement: S1 File — (PDF) [file pone.0279378.s001.pdf]

simpleISM - A straight forward guide to upgrade from confocal to ISM

Monalisa Goswami[1,2], René Lachmann[1], Robert Kretschmer[1,2], Rainer Heintzmann[1,2]

[1]Leibniz Institute of Photonic Technology;

[2]Institute of Physical Chemistry and Abbe Center of Photonics, Friedrich-Schiller University Jena

1 Works for me

Reserved DOI:
10.17504/protocols.io.kxygx9mz4g8j/v1

Monalisa Goswami

ABSTRACT

A hands-on guide to convert a confocal microscopy (here Olympus FluorView 300) into an image scanning microscopy (ISM) using a fast CMOS camera (here Andor Neo) as the detector.

PROTOCOL INFO

Monalisa Goswami, René Lachmann, Robert Kretschmer, Rainer Heintzmann . simpleISM - A straight forward guide to upgrade from confocal to ISM. **protocols.io** https://protocols.io/view/simpleism-a-straight-forward-guide-to-upgrade-from-cgeqttdw

FUNDERS ACKNOWLEDGEMENT

Abbe Centre of Photonics
Grant ID: ACP Explore

BMBF
Grant ID: 13N15713

Leibniz Center for Photonics in 208 Infection Research

CREATED

Sep 11, 2022

LAST MODIFIED

Sep 12, 2022

PROTOCOL INTEGER ID

69808

1

Replace the dichromatic beam splitter slider of the confocal microscope (Supplementary Figure

1) with a custom-made slider that has a mirror at a 45-degree angle to reflect the beam out of the confocal microscope and a hole to let the beam pass and get detected with PMT channel 2. The slider will be used to switch between confocal and ISM.

2

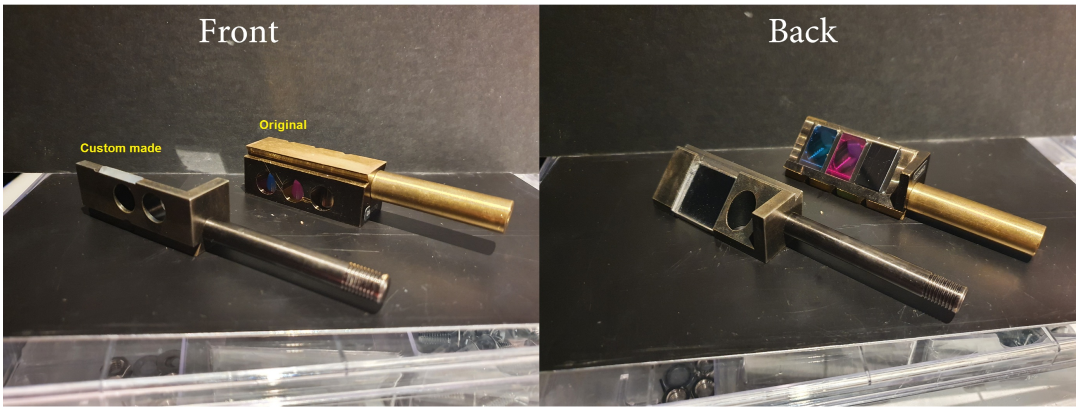

Supplementary Figure 1

3 Drill a hole in the microscope body to let the beam out of the microscope using the above-mentioned mirror slider (Supplementary Figure 2).

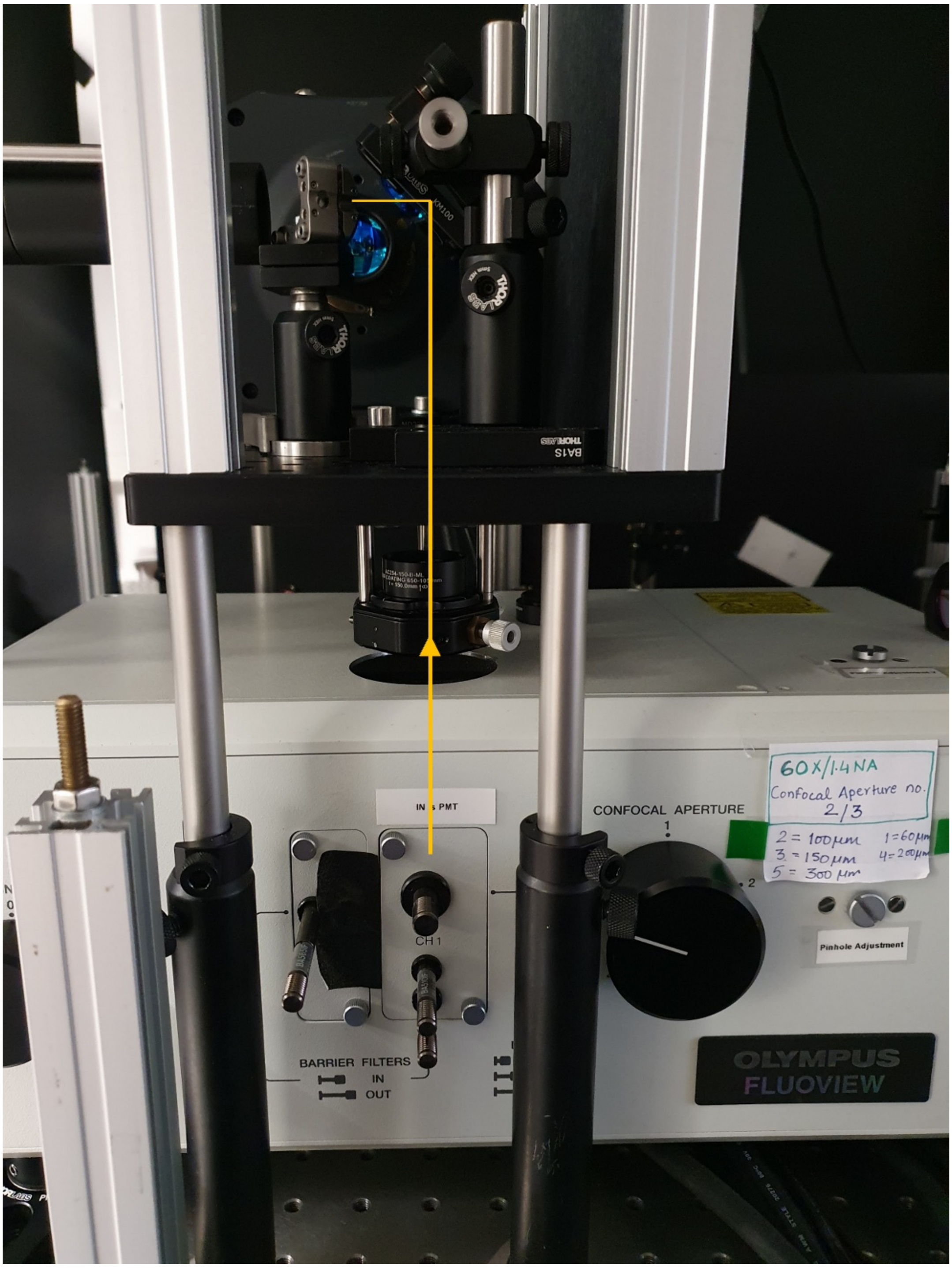

Supplementary Figure 2. Yellow line shows the beam path

4 Using a mirror slide as a sample, align the reflected laser beam onto the camera (Andor Neo sCMOS camera is used here) by running the Fluoview software in 'Point' scan mode. Use a telescope to project the pinhole plane onto the camera.

5 On the camera, select the smallest possible region of interest (ROI) (like 12x12) and align the focal spot to the centre of the ROI.

5.1 Intercepting the clock pulses from Fluoview 300 Hardware

Optional Step: The Olympus Fluoview 300 laser scanning confocal microscope has two computers, the '*power supply unit'* (PSU) and the 'trigger unit'(TU) (Supplementary Figure 3). To observe the ramp-like scan signal, connect pin 11 and pin 15 of the DB-15HD connector (situated at the back of the TU) to the. On the Fluoview software, set the scan area to '512x512' and zoom to '10'. Run the software in 'XY repeat' mode.

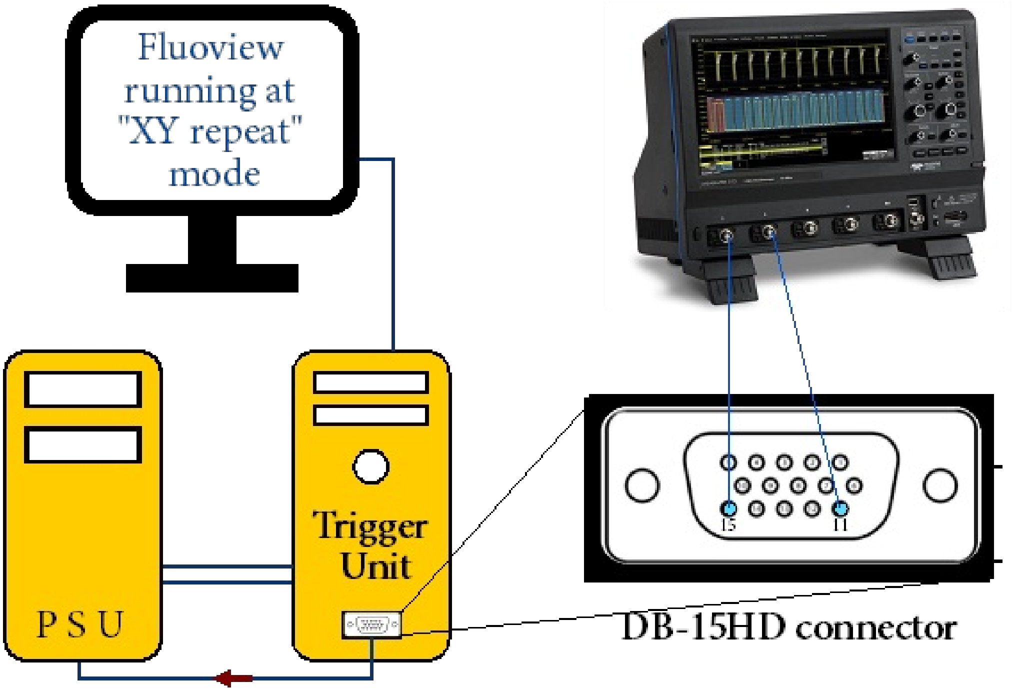

Supplementary Figure 3

Provide synchronization using Arduino Due

6 Prepare a subtractor circuit using the circuit diagram shown in *Figure 3* in the paper. Use an Arduino Due board and connect it as shown in Supplementary Figure 4.

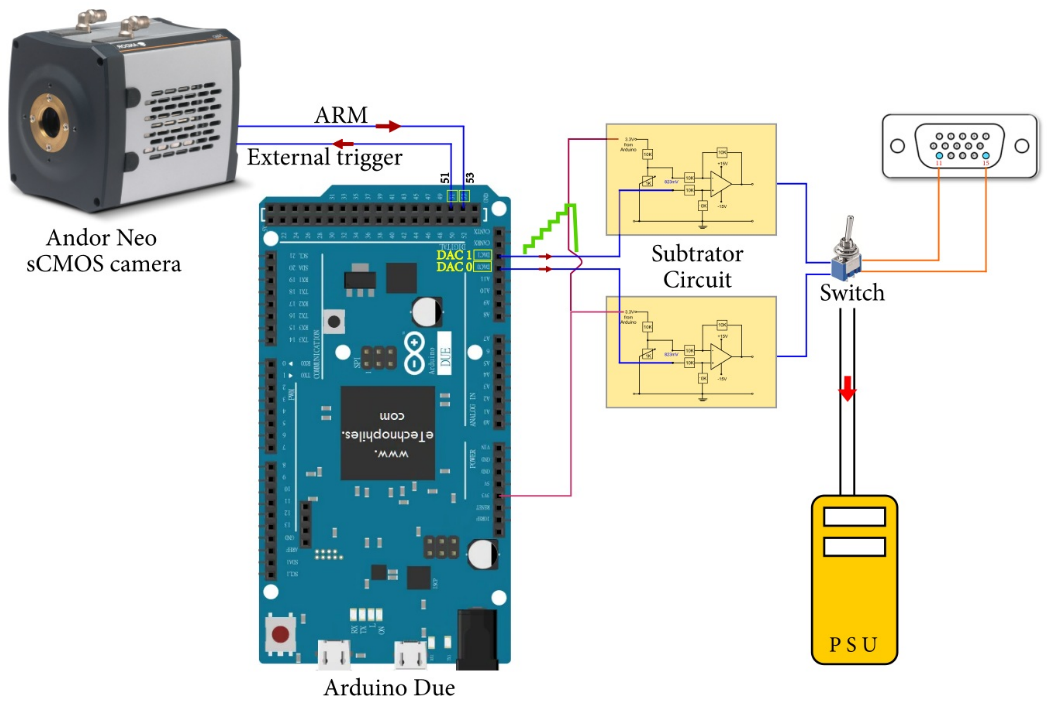

Supplementary Figure 4

Measuring ISM

7 Align the sample to the focus of the objective using the PMT detector channel 2.

8 Once the sample comes into focus, set the scan area to ‘512x512’ and zoom setting to ‘10’. Stop the scanning and toggle the switch to ISM and the slider at the mirror position.

9 Upload the provided Arduino code. Run the Fluoview software in “XY repeat” mode and acquire the camera images using the settings shown in *Table 1* in the paper.

10 Once the camera acquisition is done, stop the “XY repeat” mode in the Fluoview software and save the images on the camera in the .fits format.

11 Acquire a background image following steps 8 and 9.

12 Use the ISM reconstruction code to get the final super-resolved ISM image.
